# HIPECT4: multicentre, randomized clinical trial to evaluate safety and efficacy of Hyperthermic intra-peritoneal chemotherapy (HIPEC) with Mitomycin C used during surgery for treatment of locally advanced colorectal carcinoma

**DOI:** 10.1186/s12885-018-4096-0

**Published:** 2018-02-13

**Authors:** A. Arjona-Sánchez, P. Barrios, E. Boldo-Roda, B. Camps, J. Carrasco-Campos, V. Concepción Martín, A. García-Fadrique, A. Gutiérrez-Calvo, R. Morales, G. Ortega-Pérez, E. Pérez-Viejo, A. Prada-Villaverde, J. Torres-Melero, E. Vicente, P. Villarejo-Campos, J. M. Sánchez-Hidalgo, A. Casado-Adam, Ruben García-Martin, Manuel Medina, T. Caro, C. Villar, Enrique Aranda, M. T. Cano-Osuna, C. Díaz-López, E. Torres-Tordera, F. J. Briceño-Delgado, S. Rufián-Peña

**Affiliations:** 1 Unit of Oncologic and Pancreatic Surgery, Hospital University Reina Sofía, Menendez Pidal Av, 14004 Córdoba, Spain; 2 CIBERehd, IMIBIC, Hospital University Reina Sofía, Cordoba, Spain; 3 Unit of Surgery, Consorci Sanitari Integral, Hospital de Sant Joan Despí Moises Broggi, Barcelona, Spain; 4 Unit of Surgery, Hospital Provincial Castellón, Castellón, Spain; 5 Unit of Oncologic Surgery, Hospital Clinico Universitary Valencia, Valencia, Spain; 6 Unit of Surgery, Hospital Regional University of Malaga, Malaga, Spain; 7 Unit of Peritoneal Oncologic Surgery and Colorectal Surgery, Hospital University Nuestra Señora de la Candelaria, Tenerife, Spain; 8 Department of Surgery, Instituto Valenciano de Oncología, Valencia, Spain; 9 Surgery Department, Unit of Peritoneal Oncologic Surgery, Hospital Príncipe de Asturias, Alcalá de Henares, Madrid, Spain; 10 Unit of Oncologic and Pancreatic Surgery, Hospital Son Spaces, Palma de Mallorca, Spain; 11 MD Anderson Cancer Center, Madrid, Spain; 12 Unit of Oncologic Surgery, Hospital University Fuenlabrada, Madrid, Spain; 13 Unit of Surgery, Hospital University Infanta Cristina, Badajoz, Spain; 14 Unit of Surgery. Hospital de Torrecárdenas, Almería, Spain; 15 Department of Surgery, Sanchinarro University Hospital. “Clara Campal” Oncological Center. San Pablo University. CEU, Madrid, Spain; 16 Unit of Surgery, Hospital University Ciudad Real, Ciudad Real, Spain; 17 Unit of Pathology. Hospital University Reina Sofia, Cordoba, Spain; 18 Unit of Oncology, Hospital University Reina Sofia, Cordoba, Spain; 19 Unit of Colorectal Surgery, Hospital University Reina Sofia, Cordoba, Spain

**Keywords:** Colon carcinoma, HIPEC, Peritoneal carcinomatosis, Chemoprophylaxis

## Abstract

**Background:**

Local relapse and peritoneal carcinomatosis (PC) for pT4 colon cancer is estimated in 15,6% and 36,7% for 12 months and 36 months from surgical resection respectively, achieving a 5 years overall survival of 6%. There are promising results using prophylactic HIPEC in this group of patients, and it is estimated that up to 26% of all T4 colon cancer could benefit from this treatment with a minimal morbidity. Adjuvant HIPEC is effective to avoid the possibility of peritoneal seeding after surgical resection. Taking into account these results and the cumulative experience in HIPEC use, we will lead a randomized controlled trial to determine the effectiveness and safety of adjuvant treatment with HIPEC vs. standard treatment in patients with colon cancer at high risk of peritoneal recurrence (pT4).

**Methods/Design:**

The aim of this study is to determine the effectiveness and safety of adjuvant HIPEC in preventing the development of PC in patients with colon cancer with a high risk of peritoneal recurrence (cT4). This study will be carried out in 15 Spanish HIPEC centres. Eligible for inclusion are patients who underwent curative resection for cT4NxM0 stage colon cancer. After resection of the primary tumour, 200 patients will be randomized to adjuvant HIPEC followed by routine adjuvant systemic chemotherapy in the experimental arm, or to systemic chemotherapy only in the control arm. Adjuvant HIPEC will be performed simultaneously after the primary resection. Mitomycin C will be used as chemotherapeutic agent, for 60 min at 42–43 °C. Primary endpoint is loco-regional control (LC) in months and the rate of loco-regional control (%LC) at 12 months and 36 months after resection.

**Discussion:**

We assumed that adjuvant HIPEC will reduce the expected absolute risk of peritoneal recurrence from 36% to 18% at 36 months for T4 colon-rectal carcinoma.

**Trial registration:**

NCT02614534 (clinicaltrial.gov) Nov-2015.

## Background

Peritoneum is the second most common site of recurrence in patients with colo-rectal carcinoma (CRC) reaching a rate up to 25–35% of all recurrences [1]. The presence of peritoneal carcinomatosis (PC) is associated to fateful prognosis with a survival of 5 months if untreated and has a reported range between 5 and 15 months if treated with palliative systemic therapy, being significantly worse when is compared to survival rates after palliative systemic therapy for non-peritoneal localizations [2]. One of the risk factors identified to develop PC is the trans-serosal invasion of the tumour (pT4 _a-b_) [3, 4]. This feature represents a significant risk factor for survival by itself, in this way, the prognosis of pT4 becomes similar to patients with N2 and M1 stages with a 5 years overall survival of 20% [5]. Local relapse and peritoneal recurrence for T4 patients is estimated in 15,6% and 36,7% for 12 months and 36 months from surgical resection [6].

The effectiveness of cytoreductive surgery (CRS) and HIPEC in carcinomatosis from CRC depends on disease spread (PCI) and completeness of cytoreduction (CC), if the complete cytoreduction is achieve, the 5 years survival rates could reach between 45 and 51% in combination with HIPEC. Therefore, in selected cases with limited peritoneal carcinomatosis without distant metastasis, the CRS + HIPEC represents an attractive and defendable treatment as several phase II and III studies show that CRS + HIPEC improves the survival when is compared with systemic chemotherapy alone [7, 8].

In that sense, the use of CRS and HIPEC in early stages as proactive treatment (second-look surgery [9] or prophylactic HIPEC [10–12]) is a worthy option in the treatment of locally advanced colo-rectal carcinoma due to its promising results. Then, the use of adjuvant HIPEC associated to cytoreductive surgery in the locally advanced colo-rectal cancer (cT4) is an attractive approach from a pharmacological point of view, given the peritoneal-plasma barrier which allows for higher peritoneal cavity concentrations resulting in higher efficacy while systemic toxicity is not increased.

On the grounds of these promising results [10–12] as the theoretical benefit of prophylactic HIPEC in T4 colon cancer [6], the cumulative experience in the use of HIPEC and the minimal morbidity [13], we will conduct a randomized and controlled trial to evaluate the safety and efficacy of HIPEC with Mitomycin C which is used during surgery vs. standard treatment for high risk colon cancer (cT4) to develop peritoneal carcinomatosis [6, 14].

This study has a multicentric condition because a huge sample size is needed with a careful patients selection (cT4) [6]. Therefore, the Spanish Group of Oncological Peritoneal Surgery (GECOP) provides the correct environment to develop this ambitious study, which gives us new perspectives in the early treatment of patients with locally advanced colo-rectal cancer.

## Methods

### Objective

The purpose of this study is to determine whether Hyperthermic Intra-peritoneal Chemotherapy (HIPEC) with Mitomycin C used during surgery to treat locally advanced colorectal carcinoma is safe and effective to control the local recurrence and peritoneal carcinomatosis.

### Primary outcome measures

Loco-regional Control (LC): Time (months) elapsed from the surgical intervention to the date the patient is free of clinical and radiological loco-regional recurrence. Time frame: up to 3 years. Rate of loco-regional control % (LC%) at 12 months and 3 years will be evaluated too.

### Secondary aims are

1.- Evaluate the effect of the addition of HIPEC with Mitomycin C to cytoreductive surgery in 12 months and 3 years overall survival rate (%OS).

2.- Evaluate the effect of addition of HIPEC with Mitomycin C to cytoreductive surgery in 12 months and 3 years disease free survival rate (% DFS).

3.- Evaluate the safety (treatment-related morbidity and mortality) of addition of HIPEC with Mitomycin C to cytoreductive surgery in locally advanced colo-rectal cancer.

4.- To determine several procedural characteristics of adjuvant HIPEC such as operating time, length of hospital stay, re-admission rate, laparoscopic vs. laparotomy approach, and open vs. closed HIPEC technique.

### Design

**Fig. 1:**
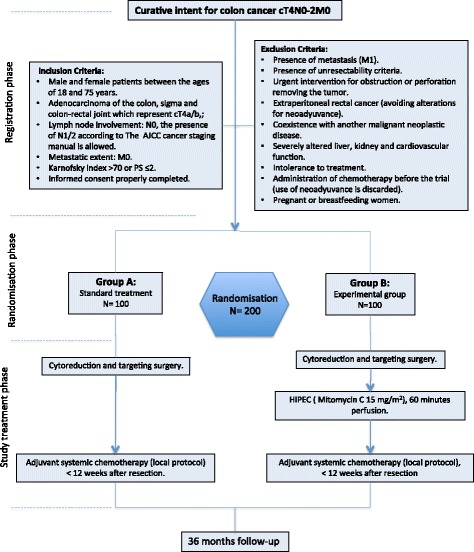
Flow-diagram HIPECT4 study

Multicentric randomized controlled clinical trial has been performed in fifteen Spanish HIPEC centres, most of them are members of GECOP (Spanish Group Peritoneal Oncologic Surgery), since November 2015. Eligible patients are randomized (in a 1:1 ratio) to cytoreduction and targeting surgery plus adjuvant HIPEC followed by standard adjuvant systemic chemotherapy in the experimental arm, or cytoreduction and targeting surgery followed by adjuvant systemic chemotherapy alone in the control arm (Fig. 1).

Adjuvant HIPEC can be performed either by open or close technique, and laparoscopic approach is allowed. Subsequently, patients will receive routine adjuvant systemic chemotherapy according to local treatment protocols within 12 weeks from HIPEC, preferably as soon as their clinical condition tolerates systemic therapy.

Follow-up will be performed routinely according to the national guideline during the first 36 months. Abdominal CT scan or abdominal MRI, tumours markers (CEA, CA19.9) will be carried out in each 6 months visit. If a suspicious of relapse is established a CT-PET, percutaneous or surgical biopsy could be performed. If the recurrent disease is developed they will be treated accordingly.

All relevant data during work up, management and follow up will be collected in an electronic case record form in a centralized way. Data will be documented in line with **‘**Good Clinical Practice**’** and Spanish legal requirements.

### Study population

Patients diagnosed with adenocarcinoma of the colon and rectum above the peritoneal reflection with cT4 N0–2 M0 stage based on preoperative imaging, are eligible for the study when they meet the following inclusion criteria:

- Male and female patients between the ages of 18 and 75 years;
- Adenocarcinoma of the colon, sigma and colon-rectal joint which represent cT4a/b in line with The American Joint Committee on Cancer: the 7th edition of the AJCC cancer staging manual;
- Lymph node involvement: N0, the presence of N1/2 according to the 7th edition of the AJCC is allowed, provided they can be resectable;
- Metastatic extent: M0.
- Karnofsky index > 70 or Performance status ≤2.
- Informed consent properly completed.

### Exclusion criteria

- Presence of metastasis (M1);
- Presence of unresectability criteria;
- Urgent intervention for obstruction or perforation removing the tumor;
- Extraperitoneal rectal cancer (avoiding alterations for neoadyuvance);
- Coexistence with another malignant neoplastic disease;
- Severely altered liver, kidney and cardiovascular function;
- Intolerance to treatment;
- Administration of chemotherapy before the trial (use of neoadyuvant is discarded);
- Pregnant or breastfeeding women.

### Treatment strategies

#### Standard care of the control arm

Treatment in the control arm of the HIPECT4 trial is the complete cytoreduction of the tumour with targeting surgery (that includes: omentectomy, hepatic round ligament, appendicectomy and bilateral oophorectomy in post-menopausian females). After resection, the adjuvant chemotherapy will be in accordance with local protocol based on CAPOX every three weeks or FOLFOX every two weeks during six months. Adjuvant chemotherapy is preferably started before 12 weeks after the primary resection.

#### Investigational treatment of the experimental arm

Treatment in the experimental arm consist of complete cytoreduction of the tumour with targeting surgery followed by HIPEC, open or close technique, with Mytomicin C. After resection the adjuvant chemotherapy will be administered as described above.

#### Hiperthermic Intraperitoneal Chemotherapy (HIPEC) procedure

After complete cytoreduction and targeting surgery by open or laparoscopic approach, adjuvant HIPEC will be administered in the experimental arm. The different types of HIPEC as open, semi-open and close technique are allowed. There is no a specific perfusion machine to perform it. The duration of HIPEC will be 60 min. The temperature must be between 42 and 43° into abdominal cavity. The dose of Mitomycin C will be 30 mg/M^2^ in a dextrose 1,5% perfusion liquid (4000cm^3^).

### Main outcome

The primary endpoint of the study is loco-regional control (LC) survival in months and loco-regional control rate % at 12 months and 3 years (LC%). The loco-regional recurrence will be evaluated by imaging tests +/− biopsy, tumour markers and/or surgical exploration.

### Secondary study endpoints

1) Peri-operative morbidity according to Common Terminology Criteria Adverse Events (CTCAE) v 4.0 and peri-operative mortality up to 30 and 90 days after surgery.

2) Overall survival (OS) in months and overall survival rate (%OS) at 12 months and 3 years.

3) Disease free survival (DFS) in months and the disease free survival rate (%DFS) at 12 months and 3 years.

### Sample size calculation

The sample size calculation has been based on loco-regional control rate (%LC) at 36 months from treatment. Experimental treatment is expected to result in a 82% loco-regional control at 36 months after surgery (absolute risk of recurrence peritoneal of 18% at 36 months) vs. the control arm that is expected to result in a 64% of loco-regional control at 36 months after surgery (absolute risk of recurrence of 36% at 36 months). To detect an absolute 18% difference in loco-regional control at 36 months a total of 190 patients (95 in each arm) is needed (error ɑ = 0.05, power = 0.80, two-sided), considering a drop-out of 5% the definitive N is 200 patients (100 in each arm).

### Data analysis

Shapiro-Wilk was used to establish the goodness of fit to the normality of the variables studied. Levene’s test was calculated to compare the equality of variances.

Continuous variables will be analysed by independent samples t-test (parametrics test) or U-Mann-Whitney (non-parametrics). Chi-square test will be used to compare qualitative variables by multiway contingency tables. For binary variables 2 × 2 tables a Chi-square with Yates correction will be used, if any frequency is ≤5 the Fisher exact test will be used.

To analyse the association between continuous variables Pearson’s correlation coefficient (parametrics) or Spearman’s correlation coefficient (non-parametrics) will be used.

Kaplan-Meier survival analysis with log rank test will be used to compare the two study groups. Every hypothesis contrasts will be two-sided. A *p*-value of ≤0.05 will be considered statistically significant.

### Safety

The safety will be evaluated as a secondary aim through the analysis of perioperative morbidity according to Common Terminology Criteria Adverse Events v 4.0 (CTCAE) and perioperative mortality at 30 and 90 days post-treatment. The medical ethical committee of the University Hospital Reina Sofia has approved the study protocol (2015–242, Ref. 2841). This study will be conducted according to the principles of the Declaration of Helsinki (Fortaleza, October 2013) and in accordance with the Medical Research Involving Human Subjects Act (WMO).

Main research will be responsible for the detection and documentation of adverse events (AE). The severity of every AE will be classified according to National Cancer Institute (NCI-CTCAE), v 4.0, with 5 grades of severity and will be registered in the Data Common Registry (DCR). This study will meet with all local legal requirements. Additionally, it will meet all the requirements of ICH Guideline for Clinical Safety Data Management, Definitions and Standards for Expedited Reporting, Topic E2A.

## Discussion

### Rationale for HIPECT4 design

The objective of HIPECT4 study is to evaluate the role of HIPEC in the prevention of peritoneal recurrence in patients with T4 colo-rectal carcinoma. Trans-serosal invasion or local organs infiltration (T4) increases the absolute risk to develop peritoneal carcinomatosis after surgical resection (17–50%) [4–6, 15]. It is expected that 36% of these patients will have a loco-regional or peritoneal relapse at 36 months after resection [6]. The hypothesis of our study is that the prophylactic HIPEC using Mitomycin C would reduce the absolute risk of peritoneal recurrence up to 18% at 36 months with a significant effect in the patient survival.

In a recent systematic review [10] seven comparative studies and five cohort studies were selected. The treatment schedules varied from repeated fluoropyrimidine-based IPEC administration in an ambulatory setting to intra-operative (HIPEC) procedures using Mitomycin-C or Oxaliplatin. The reported rates of major complications related to adjuvant (HIPEC) were low. Four out of five evaluable comparative studies reported a significant difference in the incidence of PC in favour of HIPEC. All three comparative studies reporting on survival after intra-operative HIPEC showed a significant survival benefit in favour of the experimental arm. Substantial heterogeneity was observed in patient selection, treatment protocols, and treatment effect evaluation among the aforementioned studies. The most recent comparative studies, Sammartino [11] and Noura [12], which showed promising results in favour of using prophylactic HIPEC in patients with T4 colo-rectal cancer reducing the peritoneal recurrence from 28% to 4% and from 50% to 12% respectively. These studies are not randomized and controlled and there is some bias in them.

Performing a randomized controlled trial phase III to evaluate the safety and effectiveness of prophylactic HIPEC in T4 colon cancer represents an organizational challenge according to Hompes et al. [6] This is because we need a huge sample size (200 in our study design) and because of the low incidence per year of patients with these inclusion criteria in a third level hospital. It is necessary to carry out our study different centres to complete the recruitment in 2 years. The 15 hospitals included in this study have reference Units of Oncological Peritoneal Surgery, and most of them belong to the Spanish Group of Oncological Peritoneal Surgery (GECOP).

### Preoperative patients selection

The study population are patients with cT4 colo-rectal carcinoma defined by the inclusion criteria described above. The accuracy in the preoperative diagnosis and stratification by abdominal CT scan or abdominal MRI of cT4 colon cancer represents a challenge to radiologists and oncological surgeons. The preoperative diagnosis of cT4 should be performed very thoroughly and diagnosis doubts are not allowed. Those patients with a cT3/T4 confounding diagnosis will not be included in our study, because the risk to fail in the definitive stratification is high. As some patients could be classified into pT3 stage at definitive pathological results, we have taken this aspect into account in the sample size calculation. The colonoscopy with a proven colon carcinoma biopsy is necessary for inclusion.

The COLOPEC trial [15] is a multicentric randomized trial that is evaluating the use of prophylactic HIPEC in patients with high-risk colon cancer, including T4 and perforated colon. Our trial does no include neither perforated colon cancer nor urgent colectomies because we think that including different surgeons with different technical skills and different haemodynamic patient status situations could create confusion in results.

### Chemotherapeutic agent

In the HIPECT4 trial we use Mitomycin C as standard chemotherapeutic agent for HIPEC. Other similar randomized controlled trial performed by Dutch Group called COLOPEC trial uses Oxaliplatin as standard drug [15]. The use of Mitomycin C or Oxaliplatin for HIPEC in colon carcinomatosis has been widely discussed. Mitomycin C and Oxaliplatin have been frequently used as chemotherapeutic agents for HIPEC in carcinomatosis from appendiceal or colon carcinoma origin. Both agents are independent cell cycle alkylating agents and they interfere with DNA and DNA-synthesis. Because of a large molecular weight, there is a limited systemic absorption of both agents. The enhancement of cytotoxicity under hyperthermia and a maximum 2 mm tissue penetration are also comparable. Although there are no randomized studies comparing oxaliplatin and mitomycin-C for CR/HIPEC, literature suggests an equal antitumor effectiveness [16]. The advantage of oxaliplatin is the absence of neutropenia and a shorter perfusion time (30 versus 90 min.) when is compared to Mitomycin-C. The perfusion time in our study will be 60 min according to median life of Mitomycin C and according to a prophylactic procedure like this, with a dose of 15 mg/m2 [17, 18]. The Mitomicyn C has been established as a better drug than Oxaliplatin in patients with peritoneal carcinoamtosis from colorectal cancer with a low index of PSDSS [19], just like the patients of our study. Additionally, the administration of Mitomycin C for HIPEC is less complex than Oxaliplatin which requires a bidirectional therapy with intravenous 5-FU and Leucovorin. This situation could complicate the recruitment of centres for our study.

### HIPEC technique

The open technique assures optimal distribution of heat and cytotoxic solution due to manual stirring of the abdominal contents, but it has the disadvantage of heat loss (with the need of increasing the temperature of the perfusion fluid and exposing the bowel to a risk of scald injuries), the risk of leakage of cytotoxic drugs and suboptimal exposure of the anterior parietal wall. The closed technique prevents heat loss and drug spillage, it also increases drug penetration, but it does not warrant a homogeneous distribution of the perfusion fluid. As there is no superiority in OS or DFS for any technique, in our study the HIPEC can be administered by open/semiopen colisseum or closed technique according to preferences of the centre [20].

### Surgical approach

For our study the laparoscopic approach is allowed if the oncological principles are respected. The laparoscopic approach for colon resection has demonstrated similar oncological results when it is compared with open approach [21]. The different teams must respect the two principles of our study: the first one is the completeness of cytoreduction of the primary tumour and the target organs and second is the administration of HIPEC with Mitomycin C 42–43° during 60 min by open or closed technique.

We have chosen the prophylactic resection of target organs (risk of harbouring tumour cells), such as omentectomy, appendicectomy and oophorectomy in postmenopausians females, like in the Sammartino et al. [12] study, in addition to adjuvant HIPEC. Although potential micro metastases in these sites are sufficiently treated with HIPEC, half of the patients in our study will not receive it, then, we think that the same aggressive approach with resection of target organs for every patients could decrease the incidence of loco-regional recurrence thus avoiding bias in that sense.

We have chosen to administer the HIPEC simultaneously to tumour resection, which will allow us to administer the HIPEC in one procedure and not in two different surgical procedures as COLOPEC [15] study proposes. The administration of HIPEC in a two stage could increase the morbidity and length of stay of these patients, as well as the potential delay of the usual adjuvant systemic treatment. This is taken into account in the COLOPEC discussion [15] too. Another point to discuss is about the two stage HIPEC proposed by COLOPEC study where they consider the possibility to use a laparoscopic approach for it. We think that this procedure in an emergency postoperative time could be technically difficult and the distribution of chemotherapy in the abdominal cavity could be suboptimal.

### Impact and relevance

The patients with colon carcinoma with transserosal or locally organs invasion (T4) have an increasing risk to develop metachronous peritoneal carcinomatosis (17–50%) [4–6, 15]. It is estimated that 36% of these patients will develop a loco-regional and peritoneal relapse at 36 months post-resection. According to comparative studies described above, the use of HIPEC reduces the risk of carcinomatosis between 82 and 62%. We have estimated a 50% reduction of risk. This would mean an absolute risk reduction from 36% to 18% at 36 months post-surgery. The absolute reduction of risk to develop a carcinomatosis means that in the 82% of patients would not get any benefit from the experimental treatment. This is only acceptable if the associated morbidity is relatively low which seems to be based on systematic review of the literature and our own experience [10, 18, 22].

## Conclusions

Adjuvant intra-operative HIPEC showed promising results in patients with T4 colo-rectal cancer in previous non-randomized studies. It is necessary to carry out a randomized and controlled trial to evaluate what is the role of HIPEC with mitomycin-C to prevent the loco-regional recurrence in T4 colo-rectal cancer and its impact in the survival of these patients.

## Abbreviations

5-FU: 5-fluororacil
AE: Adverse events
CAPOX: Capecitabine-oxaliplatin
CC: Completeness of cytoreduction
CRC: Colorectal carcinoma
CRS: Cytoreduction surgery
CT: Computerized tomography
DCR: Data common registry
DFS: Disease free survival
FOLFOX: 5-fluororacil-leucovorin-oxaliplatin
GECOP: Spanish group of peritoneal oncologic surgery
HIPEC: Hyperthermic intraperitoneal chemotherapy
HIPECT4: Adjuvant hyperthermic intraperitoneal chemotherapy in patients with T4 colon cancer
LC: Locoregional control
MRI: Magnetic resonance imaging
NCICTCAE: National Cancer Institute Common Terminology Criteria Adverse Events
OS: Overall survival
PC: peritoneal carcinomatosis
PCI: Peritoneal Cancer Index
PSDSS: Peritoneal surface disease severity score
